# Comparison of online adaptive and non-adaptive magnetic resonance image-guided radiation therapy in prostate cancer using dose accumulation

**DOI:** 10.1016/j.phro.2024.100662

**Published:** 2024-10-28

**Authors:** Martina Murr, Daniel Wegener, Simon Böke, Cihan Gani, David Mönnich, Maximilian Niyazi, Moritz Schneider, Daniel Zips, Arndt-Christian Müller, Daniela Thorwarth

**Affiliations:** a Section for Biomedical Physics, Department of Radiation Oncology, University of Tübingen, Germany; b Department of Radiation Oncology, University Hospital Tübingen, Tübingen, Germany; c Department of Radiation Oncology, Alb-Fils Kliniken GmbH, Goeppingen, Germany; d Department of Radiation Oncology, Berlin Institute of Health, Charité - Universitätsmedizin Berlin, Corporate Member of Freie Universität Berlin, Humboldt-Universität zu Berlin, Berlin, Germany; e German Cancer Consortium (DKTK), partner site Tübingen, and German Cancer Research Center (DKFZ), Heidelberg, Germany; f Department of Radiation Oncology and Radiotherapy, RKH-Kliniken Ludwigsburg, Ludwigsburg, Germany

**Keywords:** Dose accumulation, MR-guided Radiotherapy, MR-Linac, Deformable image registration, Prostate Cancer

## Abstract

**Background and purpose:**

Conventional image-guided radiotherapy (conv-IGRT) is standard in prostate cancer (PC) but does not account for inter-fraction anatomical changes. Online-adaptive magnetic resonance-guided RT (OA-MRgRT) may improve organ-at-risk (OARs) sparing and clinical target volume (CTV) coverage. The aim of this study was to analyze accumulated OAR and target doses in PC after OA-MRgRT and conv-IGRT in comparison to pre-treatment reference planning (refPlan).

**Material and methods:**

Ten patients with PC, previously treated with OA-MRgRT at the 1.5 T MR-Linac (20x3Gy), were included. Accumulated OA-MRgRT doses were determined by deformably registering all fraction’s MR-images. Conv-IGRT was simulated through rigid registration of the planning computed tomography with each fraction’s MR-image for dose mapping/accumulation. Dose-volume parameters (DVPs), including CTV D50% and D98%, rectum, bladder, urethra, Dmax and V56Gy for OA-MRgRT, conv-IGRT and refPlan were compared using the Wilcoxon signed-rank test. Clinical relevance of accumulated dose differences was analyzed using a normal-tissue complication-probability model.

**Results:**

CTV-DVPs were comparable, whereas OA-MRgRT yielded decreased median OAR-DVPs compared to conv-IGRT, except for bladder V56Gy. OA-MRgRT demonstrated significantly lower median rectum Dmax over conv-IGRT (59.1/59.9 Gy, p = 0.006) and refPlan (60.1 Gy, p = 0.012). Similarly, OA-MRgRT yielded reduced median bladder Dmax compared to conv-IGRT (60.0/60.4 Gy, p = 0.006), and refPlan (61.2 Gy, p = 0.002). Overall, accumulated dose differences were small and did not translate into clinically relevant effects.

**Conclusion:**

Deformably accumulated OA-MRgRT using 20x3Gy in PC showed significant but small dosimetric differences comparted to conv-IGRT. Feasibility of a dose accumulation methodology was demonstrated, which may be relevant for evaluating future hypo-fractionated OA-MRgRT approaches.

## Highlights

• Deformable dose accumulation for online-adaptive magnetic resonance guided radiotherapy (OA-MRgRT) and non-adaptive, conventional image-guided radiation therapy of prostate cancer were compared.
• Significant differences in accumulated overall doses were found for organs at risk.
• Clinical target volume coverage was similar in all approaches.
• However, estimated clinical effects were small for 20 x 3 Gy treatments.
• The developed method and workflow feasible and can be transferred to various scenarios.

## 1 Introduction

Conventional image-guided radiation therapy (conv-IGRT) is the standard of care in precise irradiation of the target volume and sparing of healthy tissue in prostate cancer (PC) treatment [1].

Currently, radiation therapy (RT) is based on planning computed tomography (pCT) images and sometimes additional magnetic resonance imaging (MRI) scans. The target volume and organs at risk (OARs) are identified and contoured on pCT and/or MRI, and the treatment dose is optimized according to clinical dosimetric criteria. Prior to irradiation, the patient’s position is validated and corrected, if necessary, based on cone-beam CT (CBCT) images acquired on the treatment couch. Correction vectors are determined via rigid registration of the CBCT to the reference anatomy defined by the pCT. Despite the precision of this targeted radiation technique, PC patients can experience urogenital side effects such as strictures, obstruction, hematuria, dysuria, and incontinence [2], [3].

With the introduction of conv-IGRT target coverage was increased, whereas the actual dose delivered to normal tissues remained highly uncertain because of volume as well as positional changes of the OARs with respect to the target. This is due to the fact that conv-IGRT lacks the ability to account for deformations and anatomical changes between fractions and changes in volume, such as bladder and rectum filling, which are critical for healthy tissue sparing [4]. Online-adaptive RT (OA-RT) techniques, such as magnetic resonance-guided radiotherapy (MRgRT), promise better sparing of OARs while ensuring high tumor dose coverage [5], as MRI scans are acquired daily, allowing for dose re-optimization and −calculation based on the daily anatomy [6].

Fractionated OA-RT necessitates deformable dose accumulation (DDA) to determine the total dose deposited in both the tumor and surrounding tissue [7]. DDA involves several steps: deformable image registration (DIR) to accommodate anatomical variations, dose mapping to determine the delivered dose to normal tissues and the target volume, and summation of deformed fractional doses to calculate the total delivered dose [8]. However, integrating DDA into the clinical workflow for OA-RT faces challenges, primarily due to uncertainties associated with DIR [9] and its technical implementation [8]. Despite these challenges, studies have demonstrated the potential of DDA in MRgRT, including predicting toxicity and facilitating treatment adaptation [5], [10], [11], [12].

The aim of this study was to investigate total deformably accumulated doses in PC after OA-MRgRT and conv-IGRT, in comparison to doses from the pre-treatment reference plan (refPlan), using relevant planning dose-volume parameters (DVPs) for clinical target volume (CTV) and OARs. The clinical significance of the differences was assessed using a normal tissue complication probability (NTCP) model.

## 2 Material and methods

### 2.1 Patient characteristics

This prospective study included data from ten PC patients who were treated with OA-MRgRT (20x3 Gy) at the 1.5 T MR-Linac (Unity, Elekta AB, Stockholm, Sweden) between March 2019 and March 2020 at the University Hospital Tübingen. The cohort’s median (range) age at treatment was 76.4 (58.8–83.1) years. All patients were part of a clinical study approved by the institutional review board (NCT02724670) and gave written informed consent. Patient inclusion criteria were histologically confirmed PC with indication for curative RT, cT1b-cT3a cN0 cM0, ECOG PS 0–2, and IPSS ≤ 12 (possibly only achieved after neoadjuvant hormone therapy or tamsulosin administration).

### 2.2 Reference planning

**Fig. 1:**
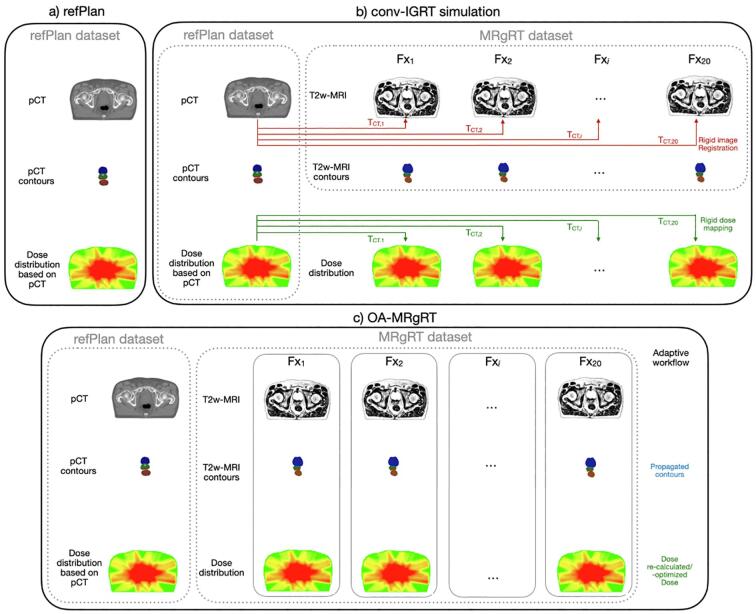
Schematic illustration of (a) the refPlan including pCT, contours and dose distribution, (b) conv-IGRT simulation with rigid registration of the pCT to each T2w-MRI and the resulting translation matrix T, and (c) OA-MRgRT with new online adaptive dose planning for each fraction.

Before treatment initiation, a refPlan was created for each patient based on the pCT (voxel size 1.17 × 1.17 × 3.00 mm^3^) (Fig. 1a), matched to a T2-weighted (T2w) simulation MRI for precise MR contouring. The radiation oncologist delineated the CTV defined by the prostate contour. The planning target volume (PTV60) was created using a 5 mm margin around the CTV (except 3 mm dorsally towards the rectum). The CTV plus up to 20 mm of the seminal vesicle base determent the CTV_SV_, as well as a respective planning target volume prescribed with 57.6 Gy (PTV57.6) with a 6 mm margin around CTV_SV_ (except 5 mm dorsally towards the rectum). Bladder, rectum, and urethra were delineated as OARs. In addition to dose prescriptions of 60.0/57.6 Gy, maximum dose constraints for rectum, bladder, and urethra were set to Dmax ≤ 61.0 Gy. Further OARs constraints were V56Gy < 13.5 % for the rectum and V56Gy < 18.0 % for the bladder. Dose planning and calculation was performed on a voxel grid size of 3 × 3 × 3 mm^3^, employing nine IMRT beams with the MONACO treatment planning system (Version 5.51.11, Elekta AB, Stockholm, Sweden).

### 2.3 Conventional-IGRT workflow simulation

Before each daily radiation fraction using MRgRT, a T2w-MRI scan (voxel size 0.83 × 0.83 × 2.00 mm^3^) was acquired. To simulate a conv-IGRT workflow, the pCT was rigidly registered to the daily T2w-MRI of each fraction (cf. Fig. 1b) using a bounding box around the CTV taking into account translations only. After rigid image registration, the refPlan dose was mapped accordingly to each fraction’s T2w-MRI.

### 2.4 Online adaptive MRgRT

OA-MRgRT was applied based on the daily acquired T2w-MRI. For each fraction, the refPlan was adapted to the patient's anatomy using the adapt-to-shape (ATS) or, in some cases, the adapt-to-position (ATP) workflow (cf. Fig. 1c and Table 1). ATP was only used after visual inspection by a radiation oncologist and no relevant anatomical and position changes after initial image registration.

**Table 1:** Summary of the OA-MRgRT workflows used per patient. Fraction numbers using ATS or ATP based on the pCT or previous T2w-MRI are given.

Patient	ATS	ATP	
		to the pCT scan	to a T2w-MRI of a previous treatment
P01	17	3	0
P02	9	11	0
P03	19	1	0
P04	20	0	0
P05	12	7	1
P06	14	6	0
P07	8	12	0
P08	16	3	1
P09	17	3	0
P10	13	0	7
Total	**145**	**46**	**9**

In the ATS workflow, the pCT and contours were deformably registered to the current T2w-MRI. Contours were propagated, corrected, or re-delineated, if necessary, by the radiation oncologist. Each adaptive plan was re-optimized based on the respective T2w-MRI and the new fraction contours using a synthetic CT with assigned bulk electron density [13]. For further details regarding the workflows, please refer to [6]. In contrast, if the ATP workflow had been used, the daily T2w-MRI was rigidly aligned with either the pCT or an individually selected T2w-MRI from a prior treatment fraction. The location of the isocenter was updated accordingly, and the planned dose of the respective fraction was then recalculated for this position.

### 2.5 Dose mapping and accumulation

For the DDA procedure and analysis, the contours of the CTV created during clinical OA-MRgRT treatment on the daily T2w-MRI were used after cross-checking by an experienced radiation oncologist, who also delineated the urethra on each T2w-MRI manually. Bladder and rectum contours were created on the T2w-MRIs using automatic contouring (ADMIRE auto contouring, V1.0, Elekta AB, Stockholm, Sweden) to ensure high anatomical accuracy of the DDA analysis.

**Fig. 2:**
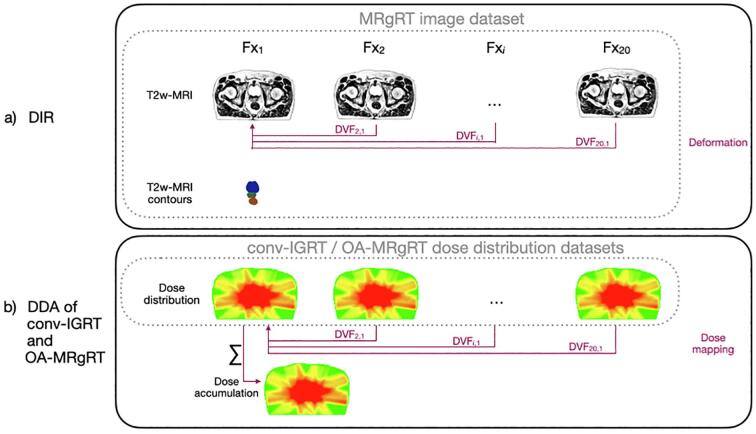
Schematic overview of a) the DIR and b) DDA procedure for conv-IGRT and OA-MRgRT. The DIR resulted in a DVF used for dose mapping for both accumulation approaches rigid registered refPlan doses of conv-IGRT and the daily adaptive doses of OA-MRgRT.

As a first step of DDA for both, conv-IGRT and OA-MRgRT, the daily T2w-MRIs of the OA-MRgRT were deformably registered to the first fraction, cf. Fig. 2a, using the auto-contours of the prostate, bladder, and rectum of the first fraction as registration guidance. Subsequently, the fraction doses were deformably mapped according to the deformation vector field (DVF) resulting from DIR (Fig. 2b). ADMIRE research (Version 3.48, Elekta AB, Stockholm, Sweden) was employed to perform DIR and dose mapping, using a hybrid intensity/structure-based algorithm [14]. The accumulation was carried out using an in-house-developed Python script (Python Version 3).

### 2.6 Analysis

The comparison between the refPlan and the two adaptive treatment strategies, namely the accumulated conv-IGRT simulation and OA-MRgRT doses, was conducted through a dosimetric analysis. This involved evaluating DVPs derived from the planning objectives used during routine plan approval in our department for the OARs rectum, bladder and urethra as well as for the prostate CTV (D98%≥57 Gy, D50%≥60 Gy). CTV_SV_ was excluded from this analysis due to the non-anatomical definition of this volume. All relevant DVPs, CTV D50% and D98%, rectum, bladder, and urethra Dmax, and rectum and bladder V56Gy, were extracted from corresponding dose-volume histograms (DVHs), computed with a bin width of 0.01 Gy.

Whether dosimetric differences between the refPlan and the two adaptive treatment strategies lead to clinically meaningful differences was calculated on the basis of the NTCP using the Lyman-Kutcher-Burman (LKB) model

$$\mathrm{NTCP}=\frac{1}{1+e^{\left(-\frac{EQD2-{\mathrm{TD}}_{50}}{m∙{\mathrm{TD}}_{50}}\right)}}$$

with EQD2 equivalent dose in 2 Gy fractions (cf. Supplementary A), TD_50_ dose at which 50 % of the population is expected to experience a complication and m slope parameter describing the steepness of the dose–response curve [15], [16], [17], [18]. The NTCP was calculated for bladder incontinence G2+ (α/β = 1.5 Gy, TD_50_ = 108.9 Gy, m = 0.24, n = 0.02) [19], and for rectum late toxicity G2+ (α/β = 3.0 Gy [20], TD_50_ = 80.8 Gy [21], m = 0.15 [15], n = 0.12 [15]).

Statistical analysis of the differences in DVPs and NTCP obtained for the different treatment approaches was carried out using the non-parametric Wilcoxon signed-rank test, with α = 0.05 and a Bonferroni correction accounting for the three different treatment approaches. Additionally, Cohen's d was calculated for the DVPs (cf. Supplementary A).

## 3 Results

Variability of bladder and rectum volumes per treatment fraction are presented in Supplementary Fig. S1. The two investigated DDA approaches, OA-MRgRT and conv-IGRT, resulted in comparable median (range) accumulated doses for CTV D50% with 60.0 (59.6–60.6) Gy and 59.9 (59.6–60.6) Gy, similar to the refPlan with 60.1 (59.7–60.6) Gy. Additionally, the approaches achieved the CTV D98% planning objective in 100 % of the patients, OA-MRgRT with a median (range) of 58.6 (57.6–59.5) Gy, conv-IGRT with 58.3 (56.9–59.4) Gy and refPlan with 58.6 (57.8–59.2) Gy. Comparing OA-MRgRT to conv-IGRT resulted in statistically significant differences for CTV D98% (p = 0.029), whereas for CTV D50% was different between conv-IGRT and refPlan (p = 0.012).

**Fig. 3:**
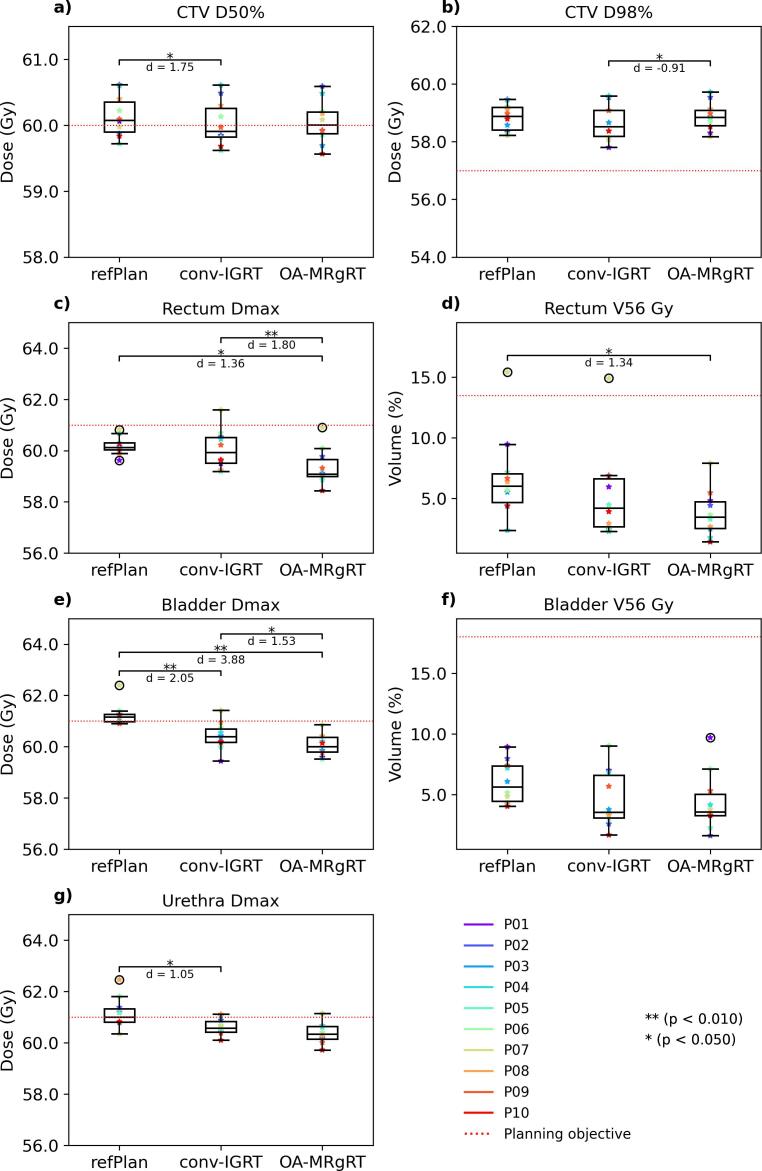
Boxplots showing the differences in the DVPs for CTV and OARs between refPlan, conv-IGRT, and OA-MRgRT. The boxes represent interquartile range (IQR), which is the range between the first quartile (Q1) and the third quartile (Q3). The black line inside the box is the median of the dataset. The length of the whiskers is set to 1.5 times the IQR. Individual data points beyond the whiskers are considered potential outliers and plotted as black circles. The color-coded points are the patient-individual results. The red dotted lines represent the planning objectives: CTV D50%≥60 Gy (a), D98%≥57 Gy (b), rectum, bladder, and urethra Dmax < 61 Gy (c, e, g), rectum V56Gy < 13.5 % (d), and bladder V56Gy < 18 % (f). Statistical analysis was performed using the non-parametric Wilcoxon signed-rank test, with α = 0.05 and Bonferroni correction accounting for the three different treatment approaches, in addition to Cohen's d.

**Fig. 4:**
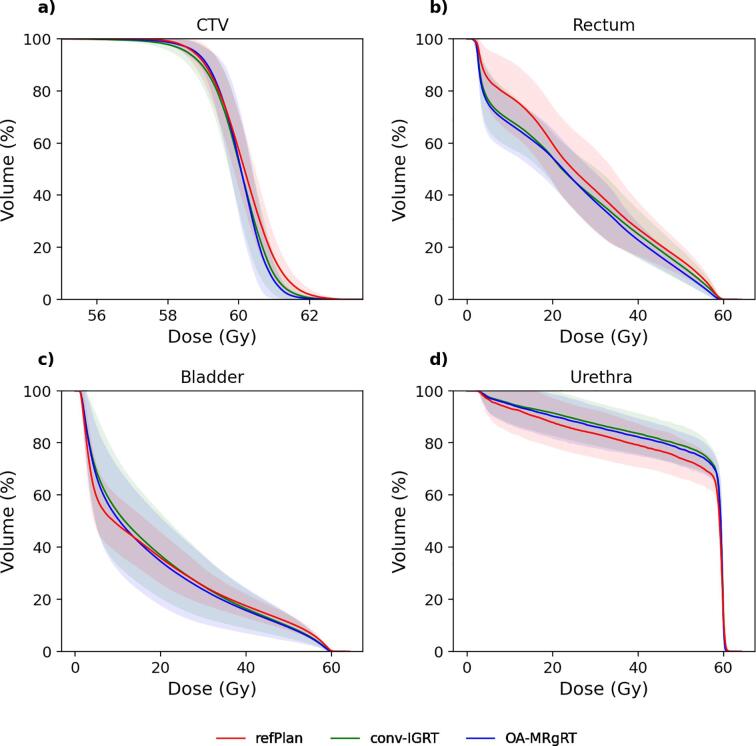
Illustration of the mean DVHs results across all patients for refPlan (red), conv-IGRT (green) and OA-MRgRT (blue) in the CTV (a), rectum (b), bladder (c), and urethra (d). The transparent area for each approach represents the standard deviation across all patients.

**Fig. 5:**
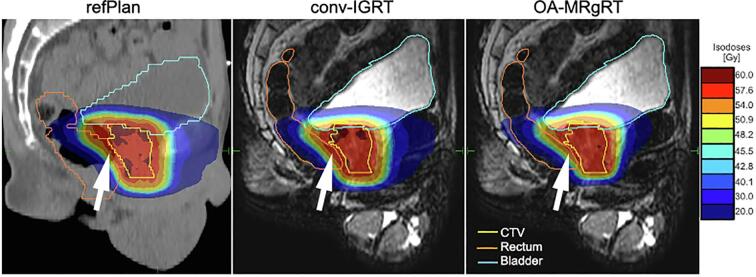
Sagittal view of the refPlan and the different accumulated dose distribution results, conv-IGRT and OA-MRgRT, for patient 10. The contours of CTV, rectum, and bladder are shown.

In compliance with the planning objectives defined for OARs, the rectum Dmax was attained for 10/10 patients for both the OA-MRgRT and the refPlan with a median (range) of 59.1 (58.4–60.9) Gy and 60.1 (59.6–60.8) Gy respectively, while this objective would have been reached in 90 % of the cases using conv-IGRT with 59.9 (59.2–61.6) Gy. Statistically significant differences were found between OA-MRgRT and conv-IGRT (p = 0.006) or refPlan (p = 0.012). Conversely, adherence to the rectum planning objective V56Gy was achieved with OA-MRgRT for all patients with a median (range) of 3.5 % (1.4 %-7.9 %), whereas 90 % of all patients achieved the planning constraint for conv-IGRT with 4.2 % (2.3 %-14.9 %) and refPlan with 6.0 % (2.4 %-15.4 %). Comparing OA-MRgRT and refPlan, significant statistical differences were observed (p = 0.012). The bladder Dmax planning constraint was fulfilled in 100 % of the cases by OA-MRgRT, 90 % by conv-IGRT, and 30 % by refPlan, with a median (range) of 60.0 (59.5–60.9) Gy, 60.4 (59.4–61.4) Gy and 61.2 (60.9–62.4) Gy, respectively. Additionally, bladder Dmax demonstrated significant differences comparing OA-MRgRT and conv-IGRT to refPlan (both p = 0.006) and OA-MRgRT to conv-IGRT (p = 0.018). However, all approaches achieved 100 % for the V56Gy planning objective of the bladder with a median (range) 3.6 % (1.6 %-9.7 %) for OA-MRgRT, 3.5 % (1.7 %-9.0 %) for conv-IGRT, and 5.6 % (4.0 %-8.9 %) for refPlan. For the urethra Dmax, attainment levels were 90 %, 80 %, and 50 % for OA-MRgRT with a median (range) of 60.3 (59.7–61.2) Gy, conv-IGRT with 60.6 (60.1–61.1) Gy, and the refPlan with 61.0 (60.4–62.5) Gy, respectively. For urethra Dmax, a significantly lower value was observed for conv-IGRT compared to the refPlan (p = 0.029). The detailed analysis and distribution of planning constraints and DVPs for all investigated approaches are presented in Fig. 3, including Cohen's d results. Additionally, population DVHs for CTV and all OARs are visualized in Fig. 4. Fig. 5 demonstrates the resulting accumulated dose distributions of the three different approaches for a representative patient case, illustrating better rectal sparing in the high-dose area with OA-MRgRT. No significant differences were found between OA-MRgRT and conv-IGRT using DDA, with regards to NTCP (cf. Supplementary Fig. S2).

## 4 Discussion

The aim of this study was to compare DDA for two adaptive treatment strategies, conv-IGRT and OA-MRgRT, with refPlan. While the refPlan represents the intended dose for targets and OARs, a conv-IGRT approach was simulated illustrating the total accumulated dose after e.g. CBCT adaptation. In contrast, the total accumulated dose delivered to patients during OA-MRgRT including daily replanning was calculated. Our DDA study showed that OA-MRgRT allows increased OAR sparing while maintaining similar target coverage compared to the refPlan, indicating its potential clinical benefits compared to conv-IGRT. However, for the treatment schedule of 20x3Gy the overall effects and thus clinical relevance were small. Nevertheless, the established workflow will allow investigation of dosimetric differences for future hypo-fractionated treatments.

The process to generate the refPlan consists of two main phases: acquisition of the pCT including patient positioning, which typically is done a week prior to the start of the treatment, followed by contouring of targets and OARs in addition to dose planning, which require approximately one working day for the physicist and the radiation oncologist. The duration of each treatment fraction varies depending on the chosen approach. In the conv-IGRT workflow, which includes patient setup, position verification using on board imaging such as CBCT and irradiation, each treatment session takes approximately 5 min [22]. In contrast, total prostate OA-MRgRT treatment times typically range from 20 to 45 min per session, with the duration influenced by the necessity for recontouring and the speed of adaptive as well as the replanning process [23]. Throughout this time, it is crucial to reduce intra-fraction motion. Efforts to expedite the online-adaptive planning process have led to the development of automated contouring [24], [25], [26], planning [27] and dose calculation [28] tools. However, the management of intra-fraction motion during OA-RT remains challenging. To overcome this, technologies such as real time motion management were recently proposed to accommodate intra-fraction motion for patients undergoing OA-MRgRT [29], [30]. These advancements might contribute to a reduction of the overall OA-MRgRT treatment time in the future.

In this study, the decision to analyze CTV planning constraints instead of PTV after DDA was based on the clinical rationale to ensure CTV dose coverage by defining an artificially constructed PTV contour [31]. Consequently, analyzing planning objectives, particularly CTV D50% and D98%, offers valuable insights into the dosimetric performance of treatment planning and delivery after DDA. Our findings demonstrated that both approaches, OA-MRgRT and conv-IGRT achieved similar median CTV doses compared to the refPlan, indicating consistent target coverage across the different approaches. This consistency suggests that, irrespective of the specific treatment approach employed in our study, maintaining a comparable level of target coverage was prioritized to ensure similar tumor control.

In the assessment of OAR-DVPs, differences were found between refPlan, conv-IGRT, and OA-MRgRT, particularly in rectum, bladder, and urethra. Although the OAR planning constraints were not fully achieved for the refPlan across patients, OA-MRgRT effectively met these constraints in all but one OAR-DVPs (Dmax urethra). The comparison between OA-MRgRT and conv-IGRT showed a decrease in median high dose constraints using OA-MRgRT. This indicates that OA-MRgRT may be more effective at sparing OARs due to its ability to adapt the treatment plan for each fraction based on daily high resolution anatomical imaging feedback, in contrast to conv-IGRT, where the plan is based on a single pre-treatment image. We hypothesize that DDA considering multiple fractions results in smoothing high dose tails of DVHs and thus leads to reduced maximum OAR-DVPs. Our results are consistent with the findings of recent studies [32], [33]. However, the study by Xiong et al. [12] found that OA-MRgRT additionally enhances target coverage. These varying results highlight differing priorities in adaptive planning, specifically, the optimization of planning for target volume coverage versus minimizing OAR Dmax.

In our study, twenty fraction doses were accumulated. With a smaller number of fractions, e.g. five, the contribution of each fraction dose map will increase. Consequently, averaging of maximum DVPs will be less pronounced [34]. As a result, any discrepancies between the planned dose and the delivered dose in individual fractions may be more apparent in the accumulated dose distribution. Conversely, with a larger number of fractions, there are more opportunities for the accumulated dose to integrate and average out variations between fractions. This may result in a more uniform accumulated dose distribution, as the cumulative effect of multiple fractions tends to mitigate individual discrepancies. However, the number of fractions is not the only factor influencing the accuracy of DDA [8]. Other factors, such as the magnitude and nature of anatomical changes [35], the accuracy of DIR [9], and the robustness of the treatment planning process [36], [37], also play critical roles. On the other hand, the PACE-B trial showed that smaller fraction numbers are not inferior to the fractionation used in this study in terms of biochemical and clinical outcome [38]. Additionally, it reduces patient attendances and shortens treatment time. For DDA, this implies a requirement for robust and precise implementation and performance.

In this study, a hybrid intensity/structure-based algorithm was employed for DIR. The literature proposes two methods for dose resampling: direct dose mapping (DDM) and energy/mass transfer (EMT) [8]. We utilized the DDM method, as a recent study from our group [14] demonstrated that both methods yield comparable results. Additionally, their study indicated that the algorithm used for this study provides robust quality assurance outcomes.

The NTCP model revealed no significant differences between the two RT approaches, OA-MRgRT and conv-IGRT, and thus no clinical impact despite significant differences in the DVP evaluation. However, in this study, patients were treated with medium to long course RT applying twenty fractions of 3 Gy. We hypothesize that the difference will be higher when applying fewer RT fractions. However, further studies are needed to investigate this. Nevertheless, our study demonstrated feasibility and transferability of the established DDA-method and −workflow to various RT scenarios.

There are limitations to the study. In the OA-MRgRT workflow, anatomical changes were compensated by the online-adaptive workflow, in contrast larger anatomical deviations such as bladder and/or rectum filling which were accepted for certain treatment fractions may not have been accepted during clinical conv-IGRT using a CBCT workflow. Ultimately this may imply limitations regarding our conv-IGRT simulations. Additionally, the simulated conv-IGRT workflow did not account for any rotation during rigid registration. However, in practice, this could be compensated with a hexapod table. Moreover, the calculation of accumulated doses currently lacks error and confidence interval estimation. A further limitation is the small cohort size, which may have influenced the statistical analysis.

In conclusion, the retrospective comparison of OA-MRgRT and conv-IGRT in PC patients demonstrated feasibility and transferability of the established DDA-method and −workflow. Significant dosimetric differences were found, but due to the small effect size when applying long course treatments, these did not translate into clinical relevance with respect to NTCP. However, clinical effects might be more pronounced for hypo-fractionated RT using only few fractions.

## CRediT authorship contribution statement

**Martina Murr:** Methodology, Formal analysis, Data curation, Software, Validation, Investigation, Supervision, Visualization, Writing – original draft, Writing – review & editing. **Daniel Wegener:** Resources, Writing – review & editing. **Simon Böke:** Resources, Writing – review & editing. **Cihan Gani:** Resources, Writing – review & editing. **David Mönnich:** Resources, Writing – review & editing. **Maximilian Niyazi:** Resources, Writing – review & editing. **Moritz Schneider:** Resources, Writing – review & editing. **Daniel Zips:** Conceptualization, Funding acquisition, Resources, Writing – review & editing. **Arndt-Christian Müller:** Conceptualization, Methodology, Funding acquisition, Formal analysis, Resources, Validation, Writing – review & editing. **Daniela Thorwarth:** Conceptualization, Methodology, Funding acquisition, Formal analysis, Validation, Supervision, Visualization, Writing – review & editing.

## Declaration of competing interest

The authors declare the following financial interests/personal relationships which may be considered as potential competing interests: The author Daniela Thorwarth is an Editor-in-Chief for Physics and Imaging in Radiation Oncology and was not involved in the editorial review or the decision to publish this article. MM and DT report institutional collaborations including financial and non-financial support by Elekta AB, Philips, TheraPanacea, Dr. Sennewald, Brainlab and PTW Freiburg. MM, ACM, CG, DT and DZ acknowledge funding through the German Research Council (DFG), grants no. MU 4603/1-1 (PAK997/1). MM acknowledges funding through the German Research Council (DFG), grants no. ZI 736/2-1. All other authors do not declare financial interests/personal relationships.
