# Supplementary material for: Comparison of online adaptive and non-adaptive magnetic resonance image-guided radiation therapy in prostate cancer using dose accumulation
Source: Phys Imaging Radiat Oncol. 2024 Oct 28;32:100662. doi: 10.1016/j.phro.2024.100662 (PMC11564916; doi:10.1016/j.phro.2024.100662)
Supplement: Supplementary Data 1 [file mmc1.docx]

Supplementary material

**Supplementary A: Material and Methods, Analysis**

EQD2 was calculated with the linear-quadradic model

$$EQD2=\left( \frac{\sum_{i} (D_{i}^{n}\cdot V_{i})}{\sum_{i} V_{i}} \right)^{\frac{1}{n}}\cdot\frac{d+\frac{\alpha}{\beta}}{2+\frac{\alpha}{\beta}}$$

where *D_i_* specifies the dose and *V_i_* the volume at dose level *i*, respectively. Parameter *n* describes the volume effect, *d* the dose per fraction and the α/β ratio the dose-response relationship.

Cohen's *d* was calculated by

$$d=\frac{M_{1}-M_{2}}{s_{p}}$$

with *M_1_*​ and *M_2_​* being the mean values of the two groups to be compared and *s_p_* the pooled standard deviation which is a weighted average of the standard deviations of the two groups.

**Supplementary B: Results**


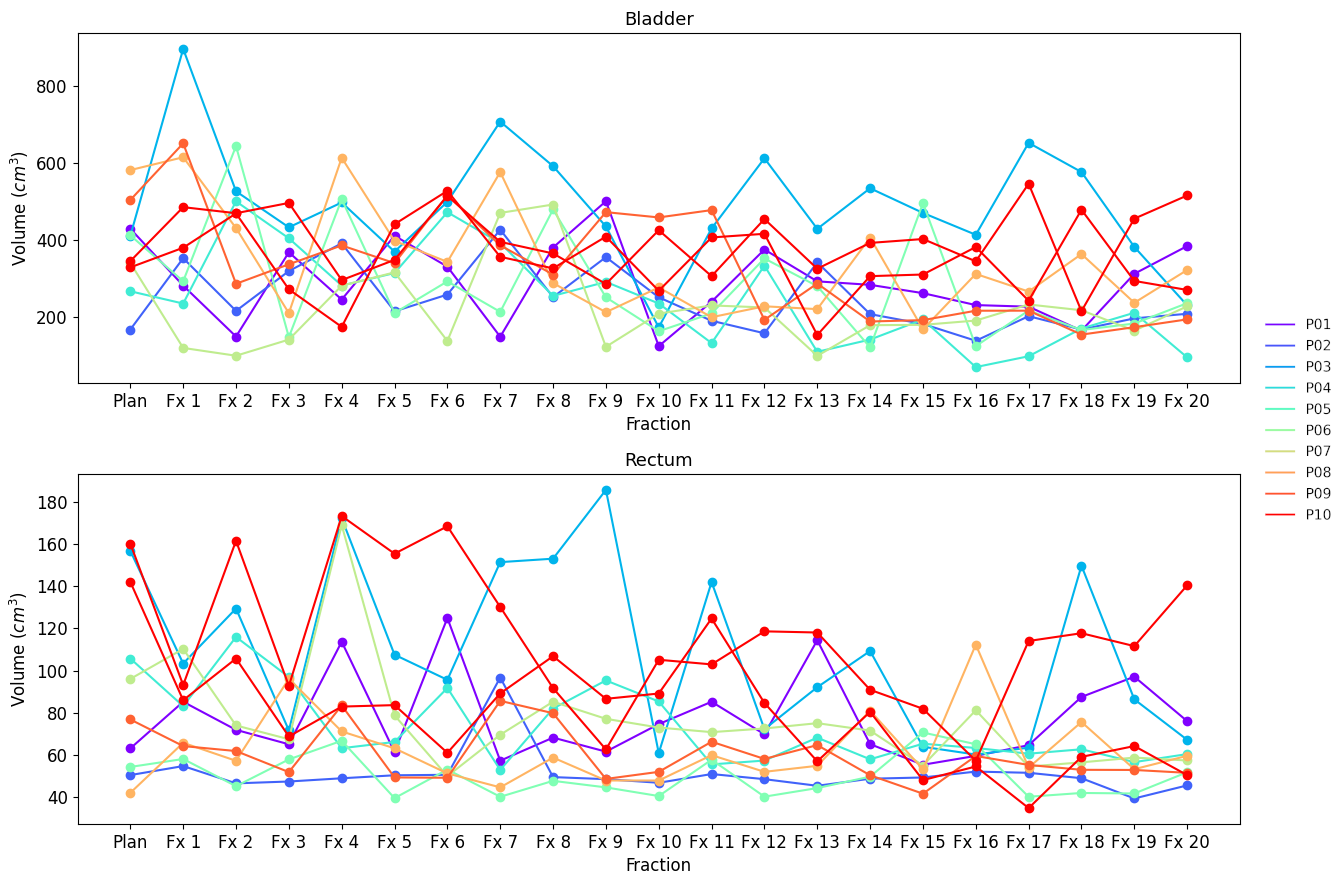


*Supplementary Figure S1: Variability of bladder and rectum volumes for the plan and across fractions per patient.*

The two investigated DDA approaches, OA-MRgRT and conv-IGRT, yielded comparable median (range) NTCP results (cf. Supplementary Figure B.2), in detail for bladder incontinence G2+ with 2.4% (2.2-3.0%) and 2.4% (2.3-2.9%) and for rectum late toxicity G2+ OA-MRgRT and conv-IGRT resulted in 0.4% (0.4-0.6%) and 0.5% (0.4-0.7%). The statistical analysis yielded no significant differences.


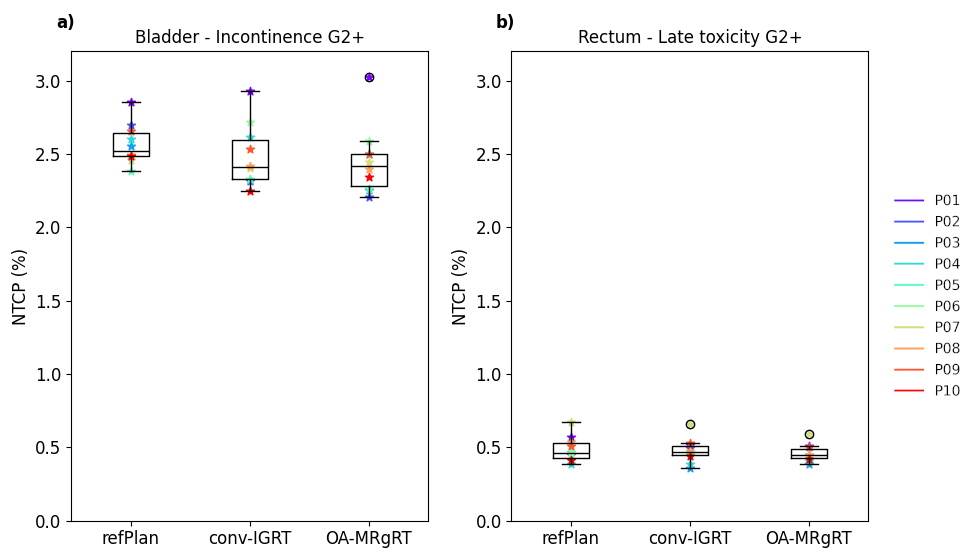


*Supplementary Figure S2: Boxplots showing the differences in the NTCP for a) bladder incontinence G2 and b) rectum late toxicity G2+ between refPlan, conv-IGRT, and OA-MRgRT. The boxes represent interquartile range (IQR), which is the range between the first quartile (Q1) and the third quartile (Q3). The black line inside the box is the median of the dataset. The length of the whiskers is set to 1.5 times the IQR. Individual data points beyond the whiskers are considered potential outliers and plotted as black circles. The color-coded points are the patient-individual results.*
